# Hydrogen water intake via tube-feeding for patients with pressure ulcer and its reconstructive effects on normal human skin cells *in vitro*

**DOI:** 10.1186/2045-9912-3-20

**Published:** 2013-09-10

**Authors:** Qiang Li, Shinya Kato, Daigo Matsuoka, Hiroshi Tanaka, Nobuhiko Miwa

**Affiliations:** 1 Department of Radiological Technology, Faculty of Health Sciences, Butsuryo College of Osaka, Otorikitamachi 3-33, Nishi-ku, Sakai, Osaka 593-8328, Japan; 2 Life Science Research Center, Mie University, Kurimamachiya-cho 1577, Tsu, Mie 514-8507, Japan; 3 Hiroshima Kasei Co. Ltd, Matsuhama Machi 2-2-11, Fukuyama, Hiroshima 720-0802, Japan

**Keywords:** Hydrogen-dissolved water, Pressure ulcer, Wound healing, Type-I collagen, Reactive oxygen species

## Abstract

**Background:**

Pressure ulcer (PU) is common in immobile elderly patients, and there are some research works to investigate a preventive and curative method, but not to find sufficient effectiveness. The aim of this study is to clarify the clinical effectiveness on wound healing in patients with PU by hydrogen-dissolved water (HW) intake via tube-feeding (TF). Furthermore, normal human dermal fibroblasts OUMS-36 and normal human epidermis-derived cell line HaCaT keratinocytes were examined *in vitro* to explore the mechanisms relating to whether hydrogen plays a role in wound-healing at the cellular level.

**Methods:**

Twenty-two severely hospitalized elderly Japanese patients with PU were recruited in the present study, and their ages ranged from 71.0 to 101.0 (86.7 ± 8.2) years old, 12 male and 10 female patients, all suffering from eating disorder and bedridden syndrome as the secondary results of various underlying diseases. All patients received routine care treatments for PU in combination with HW intake via TF for 600 mL per day, in place of partial moisture replenishment. On the other hand, HW was prepared with a hydrogen-bubbling apparatus which produces HW with 0.8-1.3 ppm of dissolved hydrogen concentration (DH) and −602 mV to −583 mV of oxidation-reduction potential (ORP), in contrast to reversed osmotic ultra-pure water (RW), as the reference, with DH of < 0.018 ppm and ORP of +184 mV for use in the *in vitro* experimental research. In *in vitro* experiments, OUMS-36 fibroblasts and HaCaT keratinocytes were respectively cultured in medium prepared with HW and/or RW. Immunostain was used for detecting type-I collagen reconstruction in OUMS-36 cells. And intracellular reactive oxygen species (ROS) were quantified by NBT assay, and cell viability of HaCaT cells was examined by WST-1 assay, respectively.

**Results:**

Twenty-two patients were retrospectively divided into an effective group (EG, n = 12) and a less effective group (LG, n = 10) according to the outcomes of endpoint evaluation and the healing criteria. PU hospitalized days in EG were significantly shorter than in LG (113.3 days vs. 155.4 days, *p* < 0.05), and the shortening rate was approximately 28.1%. Either in EG or in LG, the reducing changes (EG: 91.4%; LG: 48.6%) of wound size represented statistically significant difference versus before HW intake (*p* < 0.05, *p* < 0.001). The *in vitro* data demonstrate that intracellular ROS as quantified by NBT assay was diminished by HW, but not by RW, in ultraviolet-A (UVA)-irradiated HaCaT cells. Nuclear condensation and fragmentation had occurred for UVA-irradiated HaCaT cells in RW, but scarcely occurred in HW as demonstrated by Hoechst 33342 staining. Besides, under UVA-irradiation, either the mitochondrial reducing ability of HaCaT cells or the type-I collagen construction in OUMS-36 cells deteriorated in RW-prepared culture medium, but was retained in HW-prepared culture medium as shown by WST-1 assay or immunostain, respectively.

**Conclusions:**

HW intake via TF was demonstrated, for severely hospitalized elderly patients with PU, to execute wound size reduction and early recovery, which potently ensue from either type-I collagen construction in dermal fibroblasts or the promoted mitochondrial reducing ability and ROS repression in epidermal keratinocytes as shown by immunostain or NBT and WST-1 assays, respectively.

## Introduction

PU is common in the immobile elderly or other immobile patients suffering from diseases such as spinal cord injury, amyotrophic lateral sclerosis, multiple sclerosis, and muscular dystrophy, etc. Furthermore, aged and weak bedridden patients belong to a high risk population for PU [1]. It is estimated that there are over one million elderly people who are suffering from the skin peculiarity and are facing the risk factors of PU in USA [2]. Fundamentally, it is usually pointed out that social, psychological and financial expenses for PU are immeasurable, patients and their families as well as health care providers are always receiving the mental strain [3].

For PU, it is a primary research task to explore a cheap but effective preventive and curative method. Although various methods for prevention and treatment have been developed, they are far from sufficiently succeeding. While slightly delayed, basic studies are seen to steadily proceed in the same way as the clinical study. As the basic studies for wound healing, a lot of researchers are focusing on skin-constructing proteins such as collagen, elastin, laminin and fibronectin, and on metabolic activity and proliferating ability of dermal fibroblasts [4,5].

In relation to this matter, we had confirmed the fact that HW, as an external use for skin, can promote the construction of the type-I collagen in fibroblastic cells of dermis [6-8]. We prepared HW with a hydrogen-bubbling apparatus, exhibited a DH of 1.13 ppm and an ORP of −741 mV, in contrast a DH of < 0.01 ppm and an ORP of +150 mV for normal water [6]. Simultaneously, normal human dermal fibroblasts OUMS-36 and normal human epidermis-derived keratinocytes HaCaT were cultured using an immunostain, in addition, WST-8 and DAPI stains were conducted to examine the cytoprotective effects of HW against UVA-ray irradiation. Six Japanese subjects were enrolled in a trial of HW-bathing (DH, 0.2-0.4 ppm) every day for 3 months. The results obtained showed that HW-bathing significantly improved wrinkles on the back of the neck in four subjects on 90th day as compared to day 0. Thus the conclusion was achieved, in which HW can serve as a daily skin care routine to repress UVA-induced skin damages by ROS-scavenging and promotion of type-I collagen synthesis in dermis. On the other hand, many basic research studies demonstrated that HW is widely applied to various diseases, as an oral intake for absorbing via the gastrointestinal tract [9-14]. The researches obviously suggest that whether using a bathing type or oral intake type of treatment, HW is still an effective method to repair the skin and scavenge the ROS [15-17].

We theorized that a routine care treatment in combination with HW intake via TF for patients with PU may improve wound healing and maintain a better health condition than before. The purpose of this study is to clarify the clinical effectiveness of wound healing for patients with PU by means of an intake of HW via TF. Furthermore, OUMS-36 cells and HaCaT cells were examined to analyze the mechanisms relating to whether hydrogen plays a role in wound healing at the cellular level, *in vitro*.

## Methods

### Clinical materials

#### Patients

Medical record data that were analyzed for this study were obtained from twenty-two elderly Japanese patients with PU who were hospitalized and institutionalized in Kobayashi Hospital, Fukuyama City, Hiroshima Prefecture, Japan, which is a general hospital attached to a mixed long-term care facility. This study was approved by the Ethics Committee of Kobayashi Hospital.

The ages of PU patients who we treated in this study ranged from 71.0 to 101.0 (86.7 ± 8.2) years old, and ten patients were women. On the time of admission, they had suffered from one or multiple diseases and complications, and almost all of them were bedridden elderly people at a high risk of PU development, and all of them could not eat without other people’s aid. On the time of admission or during the hospitalization, all patients had been or were gradually appearing symptoms of PU.

The types of diseases and complications in these patients, not only included eating disorder but 90% also showed the prevalence of being in the aged period, and 100% had impaired mobility. However, it must be emphasized that PU incidence of new onset in Kobayashi Hospital remained approximately 2.10% in 2010–2011, persisted in low level. Because it was reported that average PU incidence was 2.43% in a nationwide survey executed by Japanese Society of Pressure Ulcers [18].

Twenty-two patients were retrospectively grouped into EG (effective group, n = 12) and LG (less effective group, n = 10) according to the outcomes of endpoint evaluation and the healing criteria. Details with regard to the discharge from hospital for whether cure or not were analyzed, and baseline data were summarized (Table 1). In data processing, results of all patients were classified as stage I-IV according to the Guideline in 2009 of EPUAP (European Pressure Ulcer Advisory Panel) & NPUAP (National Pressure Ulcer Advisory Panel) that is used as assessment for the severity of PU. Coincidentally, all patients in this study belonged to stage II or III.

**Table 1:** Characteristics of baseline data of PU patients in two groups

**Item**	**Effective group (EG)**	**Less effective group (LG)**
Number of patients	12	10
Age (mean ± SD) at onset	87.9 ± 9.0	85.5 ± 7.3
range	71.0-101.0	73.0-98.0
Gender (male/female)	4/8	8/2
Admission diagnosis		
PU	8	7
Tumor	0	1
Pneumonia	4	0
COPD	0	1
CIS	0	1
Hospitalized days (mean ± SD)	113.3 ± 89.6	155.4 ± 92.6
range	32-379	63-335
DESIGN-Rating (mean ± SD) at onset	14.0 ± 5.4	12.7 ± 3.3
Wound size (mean ± SD) at onset	6.9 ± 0.9 cm^2^	6.3 ± 0.9 cm^2^
Locations^*^		
Total	16	12
Back	3	0
Sacrum	3	5
Buttocks	3	2
Ilium	1	3
Greater trochanter	2	1
Thigh	1	0
Knee	1	0
Heel	1	1
Toes	1	0
Stages at onset (number of locations^*^)		
Stage II	6	4
Stage III	10	8

Abbreviations: *PU* pressure ulcer, *COPD* chronic obstructive pulmonary disease, *CIS* cerebral infarction sequela, *DESIGN-Rating*depth, exudate, size, inflammation/infection, granulation, necrotic tissue.

^*^Some patients had multiple locations for pressure ulcers.

### Clinical care treatments

#### Hospitalized routine care treatment

The treatment focused on preventing PU from getting worse and on restoring healthy skin. According to the routine care treatments for all patients, nonsurgical therapies were selected, such as ointment, gauze dressing, wrapping, and bed-pad were used after washing by the acidic water disinfection. The skin care, pressure relief and nutritional support were aggressively used as a part of this care treatment [1,3]. The main care steps to treat PU included:

a. Managing the tissue load.

b. Keeping the ulcer area clean and covered, and not letting it dry out.

c. Body-position changes at least every 2 hours if the patient is confined to a bed, or as often as every 15 min if sitting in a wheelchair.

d. To achieve positive nutritional nitrogen balance, patient consumed by TF approximately 30 to 35 calories per kg per day and 1.25 to 1.50 g of protein per kg per day.

#### Preparation for HW

**Figure 1:**
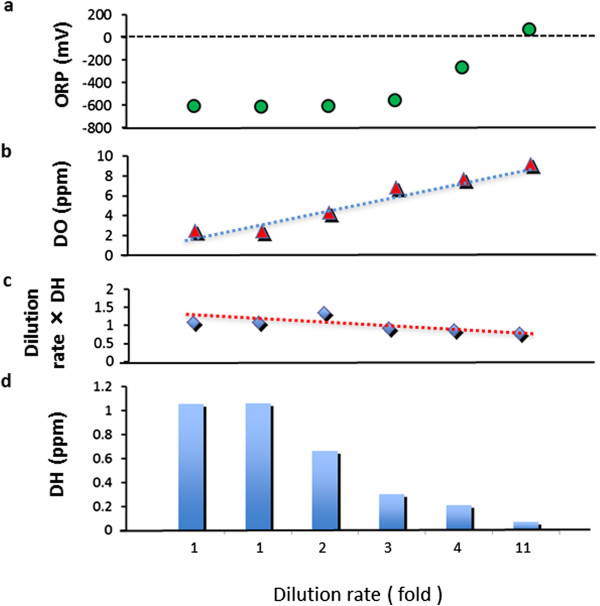
**Measurement results of diluting HW with RW.** The dilution rates are showed as Figure 1. Figures 1-**a** and -**b** show the ever-increasing tendencies on DO (dissolved oxygen concentration) and ORP (oxidation-reduction potential). Meanwhile, as shown by Figures 1-**c** and -**d**, DH (dissolved hydrogen concentration) shows the ever-decreasing tendency which indicates the dissolved hydrogen in the hydrogen water was evaporated slowly by mixing with normal regular water. On the other hand, both HW and RW have been holding the temperature range of 23.2-24.1°C and pH 7.37-7.48 no matter from 1 to 11-fold dilution rate.

**Figure 2:**
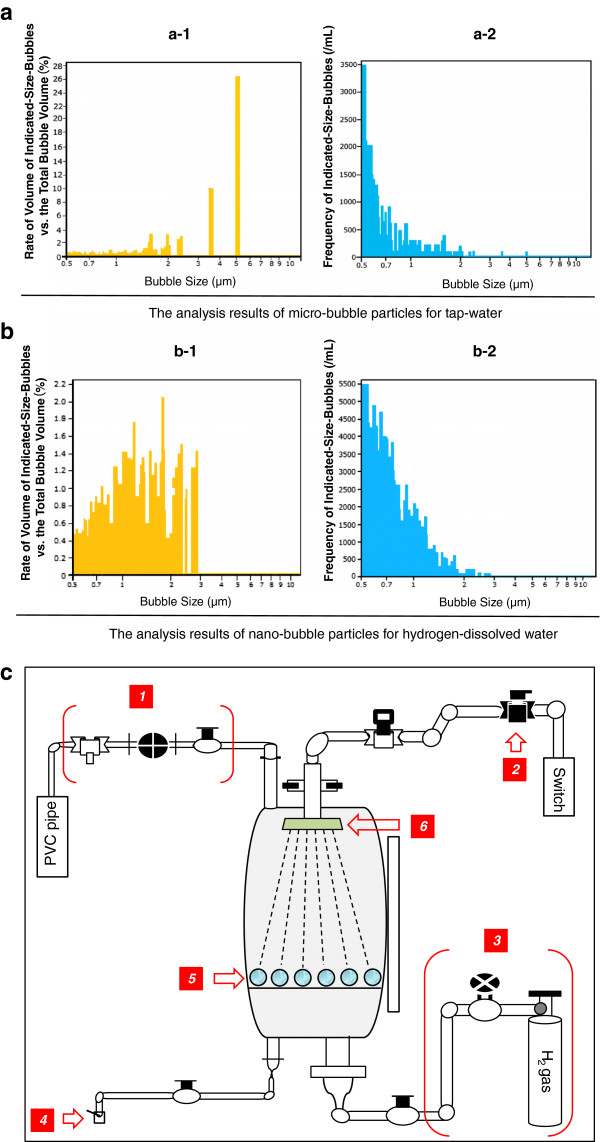
**An apparatus for manufacturing hydrogen-dissolved water and comparison of hydrogen-dissolved water with tap water from a viewpoint of their bubble particle distributions detected with an aerosol particle counter.** Figure 2-**a**: The rate of volume of indicated-size-bubbles versus the total bubble volume in tap water (dealing with the dechlorination) was analyzed by an aerosol particle counter (Beckmann-Coulter, Delsa Nano S). 2-**a-1** and **a-2**: Micro-bubble particles with diameters over 3 μm in tap water had been occupied more than 50% in the histogram. In addition, five water property parameters of tap water sample were detected as follows. DH: 0.025 ppm, DO: 8.6 ppm, ORP: +250 mV, pH: 7.30, chlorine ion concentrations: 0.015 ppm. 2-**b**: The bubble-particle distributions in hydrogen-dissolved water were analyzed by an aerosol particle counter (Beckmann-Coulter, Delsa Nano S). 2-**b-1**: Nano-bubbling particles from hydrogen-dissolved water were abundant, and in contrast, the micro-bubble particles over 3 μm were scarce. 2-**b-2**: The nano-bubble particles with diameter under 1 μm have a higher occupancy than one of the micro-bubble particles. 2-**c**: A schematic view of an apparatus (Proposal No. 2005–177724, Japan Patent) for manufacturing hydrogen-dissolved water. The mechanism means that the minimum volumes of nano size droplets are produced to maximize their surface areas, and so a high-pressure hydrogen gas was strongly incorporated into droplets inside. Apropos, the tank includes a jetting nozzle, and a guide tube of hydrogen gas which was made from the conventional device. The main accessary parts and their indices are as follows: *1. Exhaust gas section*, 0.01 Mpa; *2. Water supply section*, 0.3 Mpa, 1 Batch = 15 L/8 min; *3. Hydrogen gas supply section*, 0.9 Mpa, 0.55 L/min; *4. Outlet section of hydrogen-dissolved water*; *5. Minute particles of silica quartz porphyry*; *6. Microporous-filter hydrogen-jetting nozzle*, where the pore diameter is 6.1 μm.

HW was prepared with a hydrogen-bubbling apparatus which consists mainly of a water supply section for manufacturing RW with less 0.018 ppm of DH and +184 mV of ORP, and HW with 0.8-1.3 ppm of DH and −602 mV to −583 mV of ORP. For comparing HW with RW, the water characteristic parameters were measured with the different dilution rates (Table 2, Figures 1 and 2). It must be emphasized that some stable characteristic indicators and the proprieties for innocuity and harmlessness of hydrogen water were obtained from several separated *in vivo* and human experiments which we had reported [19-23]. Meanwhile, via tube-feeding, PU patients were enforced to intake HW of 600 mL per day, in the morning and afternoon for approximately one hour, respectively, immediately after HW was manufactured everytime.

**Table 2:** Characteristic parameters obtained from hydrogen-dissolved water vs. reversed osmotic ultra-pure water

	**DH (ppm)**	**DO (ppm)**	**ORP (mV)**	**pH**	**Water temperature (°C)**
Hydrogen-dissolved water (HW)	0.80-1.30	6.91	−602	7.40	24.1
Reversed osmotic ultra-pure water (RW)	< 0.018	8.26	+184	7.37	24.2

Abbreviations: *DH*dissolved hydrogen concentration, *DO*dissolved oxygen concentration, *ORP*oxidation-reduction potential.

### Clinical evaluations

The evaluative indices for clinical therapeutic effects on PU consisted of the hospitalized days, wound size, classifications of PU-stage and DESIGN- rating.

#### Hospitalized days

Because the increased length of hospitalized stay is an important index for a PU patient of QOL (Quality of Life), the days from admission to discharge for twenty-two patients were counted.

#### Wound size

For obtaining precise objective information and monitoring the healing degree about wound, the medical-care staff measured the size, depth and area [24], utilized photography and diagrams for recording the shape and outline of the wound.

#### Classifications of PU-stages

According to a well-known Panel Guideline established by EPUAP and NPUAP in 2009 [3], stage II includes the partial thickness for loss of skin involving epidermis, dermis or both. The ulcer is superficial and presents clinically as an abrasion or blister, but is not deeper than the dermis. On the other hand, stage III involves the full depth of the skin, and may extend into the subcutaneous tissue layer which has a relatively poor blood supply and can be difficult to heal [25,26].

#### DESIGN-rating

DESIGN was an absolute evaluation tool and consumed as a clinical indicator to assess the quality of medical care. But, its score could not be compared the severity of PU among different patients and their various ulcers. Because of this, the DESIGN-rating was invented to use as a simple and easy assessment of PU [27,28]. In our study, the DESIGN-rating score of every patient was recorded by the medical-care staff, at least once monthly.

## *In vitro* experiments

### Materials and methods

#### Normal human dermal fibroblastic cells OUMS-36

OUMS-36 cells were cultivated for 18 hours in HW- or RW-prepared Dulbecco’s modified Eagle’s medium (DMEM; Nissui Pharmaceutical Co. Ltd., Tokyo) supplemented with 10% FCS (fetal calf serum) (GIBCO) in a CO_2_ incubator to be kept at 37°C and pH 7.1-7.4 in a moistened atmosphere of 5% CO_2_. The spent medium was replaced by the fresh HW- or RW-prepared culture medium, and was at once irradiated with UVA ray at doses of 12 or 18 J/cm^2^, corresponding to the normal dose range for the human daily life. The resultant cells were stained for the nuclei with 4′,6-diamidino-2-phenylindole dihydrochloride (DAPI, Ultracruz Mounting Medium, sc-24941, Santa Cruz Biotechnology Inc., Santa Cruz, CA), and observed for type-I collagen reconstruction by immunostain using the first antibody directed against type-I collagen and the secondary antibody conjugated with FITC (fluorescein isothiocyanate), as observed with a fluorescence microscope (ECLIPSE E600, Nikon Corp., Tokyo) as previously described [6].

#### Normal human epidermis-derived keratinocytes HaCaT

HaCaT cells were similarly cultivated in HW- or RW-prepared DMEM supplemented with 10% FCS (GIBCO), and similarly UVA-irradiated. The resultant cells were examined for cell viability by WST-1 methods using (phenyl)-5-(2-disulfophenyl)-2H-tetrazolium, monosodium salt as a redox indicator, and for ROS such as superoxide anion radicals by NBT (nitro blue tetrazorium) assay as previously described [6].

## Statistical analysis

Either clinical study or *in vitro* research, the Student’s *t*-test was used to compare the difference in means ± SD between the control and treated groups using a Microsoft Office Excel 2010 software (Microsoft, Albuquerque, NM, USA) or a software package SPSS 11.0 (SPSS inc., Chicago, IL, USA) for Windows. A *p*-value that is below 0.05 was regarded to be statistically significant.

## Results

### The clinical results of routine care treatments in combination with HW via TF

#### The hospitalized days and the DESIGN-rating of PU

**Figure 3:**
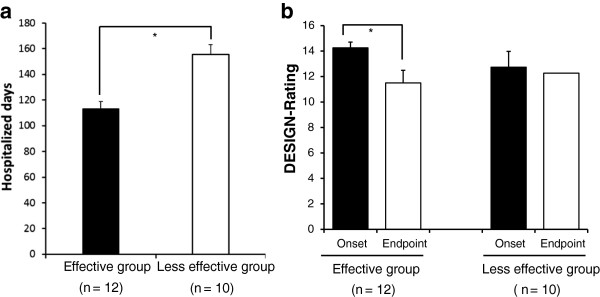
**Comparison of PU clinical effects for the hospitalized days and the DESIGN-rating in the effective group and in the less effective group.** Figure 3-**a** shows the period for the PU hospitalized days in EG was significantly shorter than in LG. Figure 3-**b** indicates that the DESIGN-rating in EG was decreased for comparing the onset with the endpoint. Pre-post evaluations were performed, where the onset and the endpoint were included. All values are statistically compared. Statistical analysis was performed using Student’s *t*-test, and the significant differences are defined as *p* < 0.05. The data are presented as the means with the standard deviation (± SD, indicated by the vertical bar). * *p* < 0.05.

For the PU patients, the hospitalized days in EG were significantly shorter than in LG (113.3 days vs. 155.4 days, *p* < 0.05), and the PU reduction rate was approximately 28.1% (Figure 3-a). Likewise, DESIGN-rating in EG was also decreased for comparing the onset with the endpoint (11.5 rates vs. 14.3 rates, *p* < 0.05) between pre-post evaluations including both in onset (evaluation in the initial time, at the day for the admission to hospital) and in endpoint (evaluation in the last time, at the day for the discharge from hospital or death day). In LG, no statistically significance was seen, in DESIGN-rating indicative of degree of severity for PU, between both of them (Figure 3-b).

#### Results of wound size in two groups

**Figure 4:**
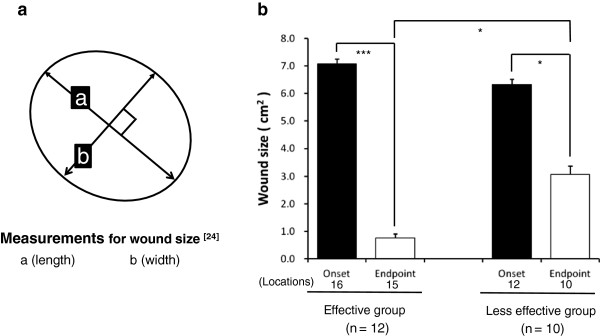
**Measurement methods for wound size and results of wound size in the wound-size reductive change between both the groups.** Figure 4-**a** demonstrates the wound measurement method. As a protocol, initially, measure the greatest length along the axial direction (head to toe), and then the greatest width along the transverse direction (side to side) using a centimeter ruler. Finally multiply distances of length and width to obtain an estimate of surface area in square centimeters (cm^2^). Figure 4-**b** indicates a statistically significant difference to the reducing change of wound size in two groups. Some patients had multiple locations for PU. Values are statistically compared. Student’s *t*-test, *p < 0.05, ***p < 0.001.

Wound measurement is an important means to know the degrees of PU, and its measuring method was demonstrated in Figure 4-a (Figure 4-a). Either in EG or in LG, the reducing changes (EG: 91.4%; LG: 48.6%) of the wound sizes represent a statistically significant difference (*p* < 0.05, *p* < 0.001). Similarly, a significant difference is also seen between both EG and LG groups (*p* < 0.05) (Figure 4-b).

#### Expression of various PU-assessment indices forcing on both stage II and stage III

**Figure 5:**
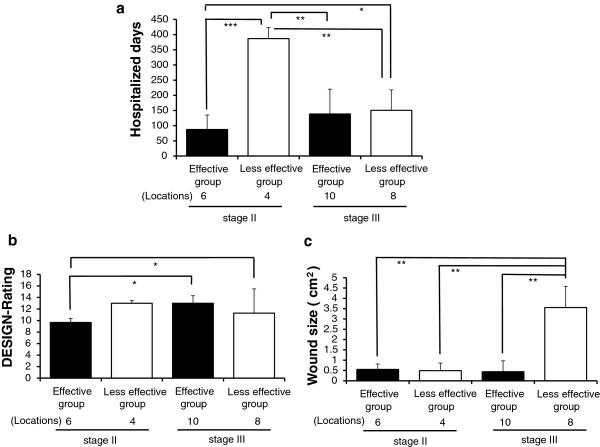
**Expression of various PU-assessment indices forcing on both stage II and stage III.** Figures 5-**a** to -**c** imply very significant differences among four subgroups based on stage II or stage III. *P*-values calculated from Student’s *t*-test, * *p* < 0.05, ** *p* < 0.01, *** *p* < 0.001.

For observing the clinical effects in many respects, including the hospitalized days, DESIGN-rating and wound size, EG and LG were subdivided to four subgroups according to classifications of PU-stages (see *Methods (3)-3*). As a result, in stage II, a period for hospitalized days in EG showed significantly shorter than in LG (87.5 days vs. 387.0 days, *p* < 0.001). Contrary to this, in two group, there was no significance statistically for hospitalized days in stage III (Figure 5-a) owing to diseases other than PU. Moreover, in EG, the DESIGN-rating obtained from subgroups of stage II and stage III depicted a statistically significant difference (*p* < 0.05) (Figure 5-b). Meanwhile, the diminishment for wound size within subgroups of stage II and stage III presents any statistical differences (Figure 5-c). In a conclusion, stage II and stage III ulcers of EG healed faster and more effectively than those of LG.

#### Results of a typical case on time-dependent wound-healing progress: for an 85-year-old female patient with PU

**Figure 6:**
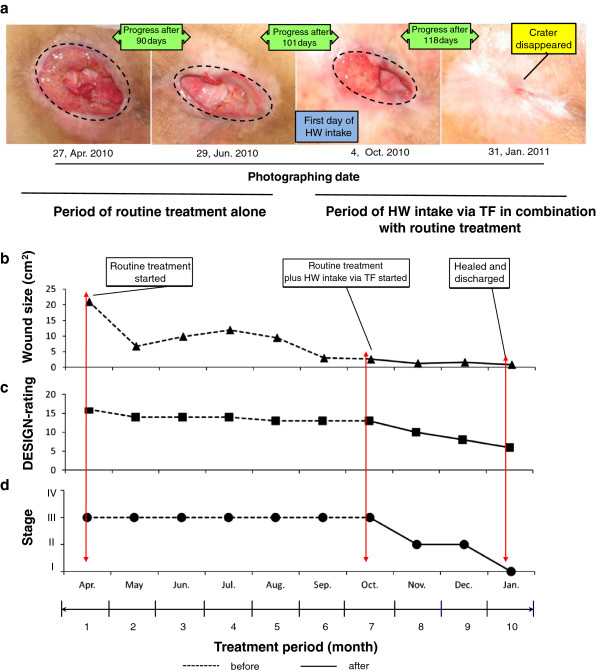
**Results of a typical case on time-dependent wound-healing progress.** An annual time-dependent wound healing progress for an 85-year-old female PU patient is reported. She was admitted to the hospital seeking treatment to PU. Figure 6-**a** demonstrates photographs for the time-dependent wound-healing progress obtained from the same patient. Figures 6-**b** to -**d** represent the decreased tendencies of wound size, DESIGN-rating, and stage, respectively.

Figure 6 showed the time-dependent wound-healing progress for an 85-year-old female patient with PU. She was admitted to the hospital for suffering from PU. Wound findings at onset included: location: sacrum; wound size (cm^2^): 20.8; stage: II; DESIGN-rating: 16. Four months after routine care treatment plus a combination with HW intake via TF, the crater nearly disappeared. Wound findings at endpoint (vs. of onset) included: wound size (cm^2^): approximately 0 (disappearance); stage: I (improve); DESIGN-rating: 6 (decrease) (Figures 6-a to -d).

#### Results of another typical case on time-dependent wound-healing progress: for an 80-year-old male patient with PU

**Figure 7:**
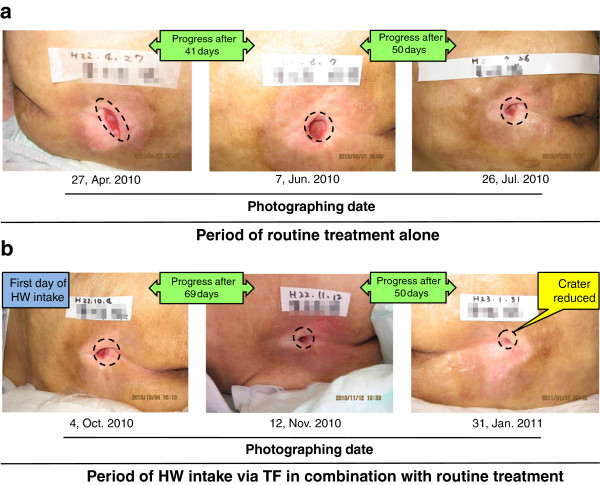
**Results of another typical case on time-dependent wound-healing progress.** Similarly to Figure 6, Figure 7 also demonstrates a time-dependent wound healing progress, for an 80-year-old male PU patient. Figure 7-**a** shows the features photographed at the former period for routine care treatment alone. On the other hand, Figure 7-**b** presents ones photographed at the latter period for routine care treatment plus HW intake. The latter period in using HW intake shows a marked improved outcome.

Figure 7 showed the time-dependent wound-healing progress for an 80-year-old male patient with PU. His hospitalized period lasted 10 months and it could be divided into the two sub-periods. During the latter 5-month period, he received HW treatment in the addition to the routine care. The outcome shows an improved result (Figures 7-a, -b).

## *In vitro* experiments

### Promotive effects on reconstruction of type-I collagen, as shown by immunostain, on normal human dermal fibroblasts OUMS-36 that were irradiated with UVA ray and then were administered with RW- or HW-prepared culture medium, respectively

**Figure 8:**
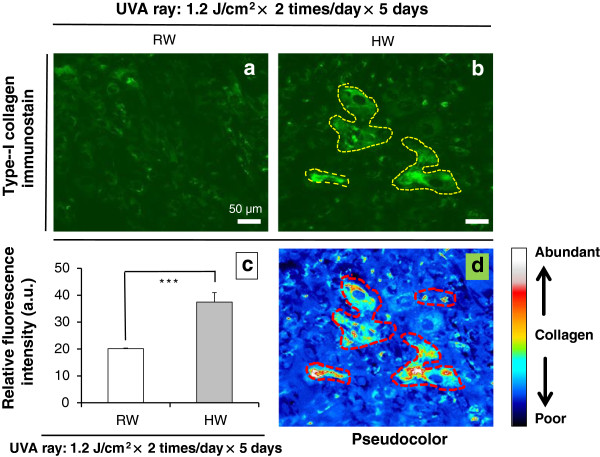
**Reconstructive effects of HW in UVA-irradiated OUMS-36 cells.** Figures 8-**a**, -**b**: Distributional expressions of type-I collagen with immunostain (green) in OUMS-36 cells that were irradiated with UVA ray and were administered with RW or HW, respectively. Figure 8-**b**: Each yellow dashed lines indicate type-I collagen-rich regions. Figure 8-**c**: Relative fluorescence intensity plotted with the ImageJ to present the pixel number. Type-I collagen stain on OUMS-36 cells that were irradiated with UVA ray and were administered with RW or HW, respectively, is showed. Figure 8-**d**: The pseudocolor feature was plotted using ImageJ as an intensity which is corresponding to type-I collagen exhibition degree per one hundred cells (μm^2^/100 cells). Magnification: ×200; scale bars = 50 μm. Student’s *t*-test, *** *p* < 0.001.

To study the reconstructive effect of HW on type-I collagen, we used immunostain on OUMS-36 cells that were irradiated with UVA ray and then were administered with RW or HW *in vitro*, respectively. Representative expressions and pixel values were plotted with a software ImageJ (http://rsb.info.nih.gov/ij/). Nuclear condensation (so-called pycnosis) and fragmentation (so-called karyorrhexis) were occurred for UVA-irradiated OUMS-36 cells in RW, but scarcely occurred in HW (Figure 8). HW group shows higher proliferation of cells with rounded morphology in fibroblasts and huge morphology, and more abundant in type-I collagen than ones of RW group.

### Proliferative effects of nucleus-DAPI stain on UVA-irradiated normal human dermal fibroblasts OUMS-36 that were administered with RW- or HW-prepared culture medium, respectively

**Figure 9:**
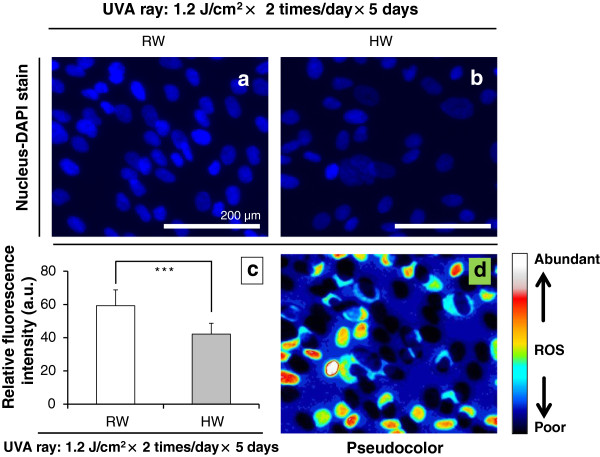
**Features of nucleus-DAPI stain on UVA-irradiated HaCaT cells.** Figures 9-**a**, -**b**: Distributional expressions of nucleus-DAPI stain (blue) in HaCaT cells that were irradiated with UVA ray and were administered with RW or HW, respectively. Figures 9-**c**, -**d**: The relative fluorescence intensity and the pseudocolor feature for type-I collagen were plotted using ImageJ. Magnification: ×200; scale bars = 200 μm. Student’s *t*-test, *** *p* < 0.001.

With fluorescence microscopy, DAPI dye can be excited by UVA ray. To examine the reconstructive effect of HW on type-I collagen by immunostain, we also counterstaind nuclei with a DAPI dye in UVA-irradiated OUMS-36 cells for observing the changes when OUMS-36 cells were administered with RW or HW *in vitro*, respectively. Representative expression and relative fluorescence intensity were plotted with the ImageJ. The facilitative effect on nuclear condensation and fragmentation was observed for UVA-irradiated OUMS-36 cells in RW, but scarcely occurred in HW as demonstrated by DAPI staining as like the result obtained from immunostain (Figure 9). Through Figure 9-c, the degrees of DAPI stain on HaCaT cells that were irradiated with UVA ray and were administered with RW or HW, respectively, were clarified.

### ROS amounts in normal human epidermis-derived keratinocytes HaCaT as quantified by NBT assay

**Figure 10:**
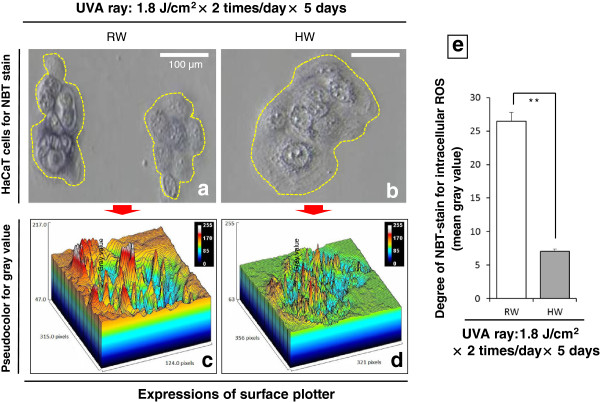
**Intracellular ROS amounts in HaCaT cells as quantified by NBT assay.** Figures 10-**a**, -**b**: The retained cell morphology and the diminished ROS were shown in HW-prepared culture medium for comparing with RW. Yellow dashed lines indicate abundant dark-blue dyes that were the reaction products where ROS such as superoxide anion radical was found to react with NBT-stain. Figures 10-**c**, -**d**: The expressions of surface plotter by ImageJ. Figure 10-**e**: The mean gray values obtained from ImageJ used to express the increase or decrease in superoxide anion radicals within normal human epidermis-derived keratinocytes HaCaT according to NBT-stain. In detail, the vertical axis shows the brightness presented as a mean gray value, which is considered as an index to show the cellular stained intensity and use to indicate ROS amounts. The cell morphologies of RW and HW were divided into the eight regions and then compared with their mean gray values by Student’s *t*-test (** *p* < 0.01). Magnification: ×200; scale bars = 100 μm.

In HaCaT cells, the intracellular ROS amounts were increased in the RW-prepared culture medium with UVA-irradiation in different UVA ray doses, but were restored in the HW-prepared culture medium as shown by NBT-stain for superoxide anion radicals. Cell morphology was observed to be more health and less harmful in HW than RW (Figure 10). Figure 10-e showed that NBT-stain was denser in dark-blue color in RW-administered cells than in HW-administered cells, indicating the intracellular ROS repression in HW-administered cells.

### Elevation of cell viability by pre-irradiational administration with hydrogen-dissolved water to UVA-irradiated HaCaT cells as assessed by mitochondrial dehydrogenase-based WST-1 assay

**Figure 11:**
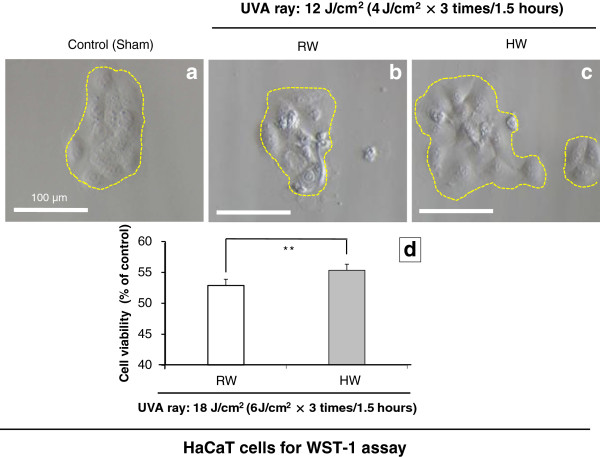
**Results of cell viability of HaCaT cells as assessed by WST-1 assay.** Figure 11-**a**: HaCaT cells are shown in the non-administered or non-UVA-irradiated status. Figures 11-**b**, -**c**: The morphologic features of HaCaT cells are shown in RW or HW, respectively, after irradiation with UVA ray. Figure 11-**d**: The cell viability is shown for HaCaT cells after UVA-irradiation by WST-1 assay. Magnification: ×400; Scale bar = 100 μm. Student’s *t*-test, ** *p* < 0.01.

In HaCaT cells, the cell viability was obviously increased in the HW-prepared culture medium with UVA-irradiation, comparing with RW-prepared culture medium by WST-1 assay (Figure 11-d). Cell morphology was also observed to be less vulnerable in terms of diverse symptoms such as cell shrinkage, nuclear condensation and cell fragmentation for HW than RW (Figures 11-b, -c). HW group showed higher proliferation of cells with rounded morphology and huge morphology, in HaCaT cells than ones of RW group. All of these evidences predicted that hydrogen-dissolved water may exert cytoprotective effects against UVA ray on HaCaT cells.

## Discussion

The purpose of the present study was to examine the clinical effectiveness of wound healing for PU using HW intake via TF. We have hypothesized that the routine care treatment in combination with HW intake for PU patients may improve wound healing, and maintain more healthy condition for them. Furthermore, normal human dermal fibroblasts OUMS-36 and normal human epidermis-derived keratinocytes HaCaT were examined to explore the mechanisms underlying to whether hydrogen plays a role in wound-healing at aspect for cutis tissue, through *in vitro* experiments.

Our clinical results seem to suggest that HW intake via TF is an effective means for wound healing of PU patients, who suffered from eating disorder. Despite the limitations caused by practicing our clinical intervention for PU, we were able to obtain the improving results in hospitalized days, wound size and other clinical indices by comparing EG with LG. Therefore, we estimated that HW absorbed by the gastrointestinal tract plays an important role in oxidative-stress reduction, extracellular matrix reconstitution, and anti-inflammatory effects. Several experiments have supported our considerations as follows.

At first, it was demonstrated that molecular hydrogen gas (H_2_) has a beneficial influence on the gastrointestinal tract [29]. Kajiya et al. established a mouse model of human inflammatory bowel disease (IBD) by supplying to mice drinking water containing a) 5% dextran sodium sulfate (DSS), b) 5% DSS and H_2_, or c) H_2_ only *ad libitum* up to 7 days. They found that on day-7, DSS-induced pathogenic outcomes including elevated levels of IL-12, TNF-α and IL-1- β in colon lesion, etc. were significantly suppressed by addition of H_2_ to DSS solution. Thereby, it was concluded that H_2_ can make an anti-inflammatory influence on gastrointestinal tract *in vivo*[30].

Secondly, Nakashima-Kamimura et al. examined whether drinking water containing the saturated dissolved hydrogen (HW: 0.8 mM H_2_ in water) is applicable by examining the effects of oxidative stress, mortality, and body-weight loss as well as serum creatinine, and blood urea nitrogen (BUN) levels. In *in vivo* experiments, their results showed that hydrogen was detected in the blood when HW was placed via gavage at a dose of 15 mL/kg in the stomach of a rat, and HW is applicable to alleviate nephrotoxic side-effects induced by an anti-cancer drug, such as cisplatin [31].

Thirdly, as molecular hydrogen gas can act as a scavenger of ROS, Cardinal et al. tested the effect of treatment with HW in a rat model of kidney transplantation. In consequence, treatment with HW improved allograft function, slowed the progression of chronic allograft nephropathy (CAN), reduced oxidant injury and inflammatory mediator production, and improved overall survival. Their conclusion was that HW is an effective antioxidant and anti-inflammatory agent *in vivo*[32].

It was previously shown that some free radicals inhibit the wound healing process [33]. H_2_ is a colorless, odorless, tasteless gas, and it possesses some peroxidant reducibility. H_2_ is possible to easily pass through the small intestine villi into the human body inside and blood stream [15], because its molecular weight is the smallest of all molecule species, and it has gaseous and electrically neutral properties, as well as it shows a strong diffusion capacity. Moreover, H_2_ maybe has its special channels for transporting into intracellular space, such as the aquaporins (AQPs) for water, especially hydrogen-holding water, and the Rhesus (Rh) proteins [34].

Thus, coupled with the body itself and the enterogenous-H_2_ presence, due to specified intestinal bacteria, HW intake via TF can play an important role on improving the formation of wound granulates on disintegrated necrosis loci, and the ability to have an anti-inflammatory effect through an ROS-reduction mechanism.

Additionally, it should be pointed out that apoptotic cells can stimulate proliferation, wound healing, and tissue regeneration [35]. We are focusing on “the apoptosis-induced compensatory proliferation” which occurs in PU [36]. Generally, necrosis has an effect of the secondary lethal damage to the wound-surrounding cells of PU through cell swelling and burst. By contrast, cell debris that is caused by cell shrinkage and fragmentation in apoptosis in which karyorrhexis (*i.e.* nuclear fragmentation) and pycnosis (*i.e.* nuclear condensation) are revealed as an early event, is subjected to endocytosis by both the migratory professional phagocytes (*e.g.* macrophages and Langerhans cells in the epidermis) and the surrounding non-professional phagocytes. So, it is thought that “the compensatory proliferation” is induced for the reason that cell debris is peaceably handled to restrain the surrounding cells within a minimal deteriorating impact. On this occasion, ROS may be able to be suppressed by the hydrogen water to evoke an apoptosis more gently, and subsequently, apoptosis that is caused in wound-surrounding cells of PU stimulates the compensatory proliferation to lead to an early healing. Indeed, Cai JM et al. reported that 2% hydrogen gas inhalation administered to a neonatal hypoxia-ischemia rat model could reduce apoptosis [37].

When HW-intake via TF was combined with routine care treatments, the wound healing process can be markedly accelerated. Hence, the effective mechanism of HW possesses at least two possible pathways, firstly is an antioxidant effect and secondly is an anti-inflammatory effect. Moreover, we thought that HW may have additional effects, *i.e.* reconstruction of collagen and cytoprotection for other dermal as well epidermal cells. Therefore, we carried out an *in vitro* experiment on normal human dermal fibroblasts OUMS-36 and normal human epidermis-derived keratinocytes HaCaT to examine their interaction. Therefore, either dermal or epidermal cells were respectively cultured in HW or RW-prepared medium. Immunostain was used for observing type-I collagen reconstruction in OUMS-36 cells and showed the promotive effect. And cell viability of HaCaT cells was examined in terms of cell morphological observation and WST-1 assay, and their generated ROS, especially superoxide onion radials, was measured by NBT assay, respectively, all of which showed the cell-death-repressive and ROS-scavenging effects.

**Figure 12:**
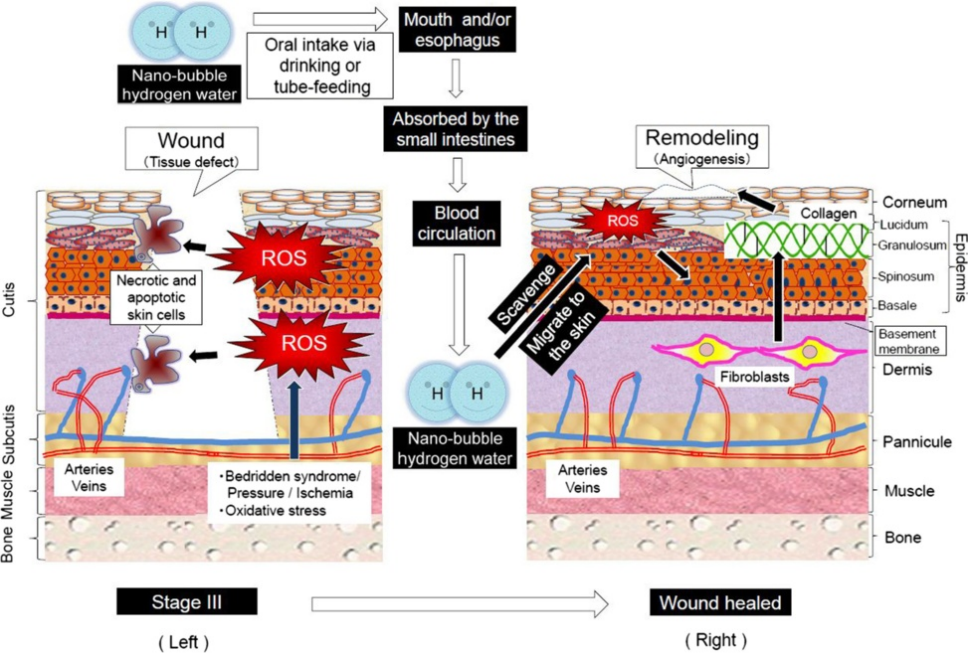
**Mechanism for wound healing of pressure ulcer by hydrogen-dissolved water.** We are predicting that ROS can lead to a pressure ulcer, and the causative process is shown by the left illustration. First of all, such diverse factors as bedridden syndrome, mechanical pressure and local ischemia produce ROS which causes necrosis and apoptosis in combination with other pathologic factors, potently resulting in wounds and tissue defects of pressure ulcer. On the other hand, the right illustration shows the healing mechanism. Oral intake of nano-bubble hydrogen water via drinking or tube-feeding passes by the mouth or esophagus, and it is absorbed by the epithelial cells of the small intestines. It is possible that hydrogen gas transpires in past from HW and inhaled by the lung. Then, the absorbed nano-bubble hydrogen migrates to cutis tissue through blood circulation and scavenges ROS abundantly generated in PU. Finally, this process results in collagen reconstruction of fibroblasts in the dermis and the proliferation of keratinocytes in the epidermis, and causes angiogenesis and remodeling for repairs in the defected tissue.

We have attempted to draw the illustrations for assuming a cure mechanism from stage III to wound healing during PU (Figure 12).

Consequently, our *in vitro* data demonstrated that intracellular ROS was diminished by HW, but not by RW, in UVA-irradiated OUMS-36 fibroblasts. Nuclear condensation and fragmentation were occurred for UVA-irradiated OUMS-36 cells in RW, but scarcely occurred in HW as demonstrated by DAPI staining. Besides, in HaCaT cells, the mitochondrial dehydrogenase, especially succinate dehydrogenase activity was diminished in RW-prepared culture medium with UVA-irradiation, but was retained in HW-prepared culture medium as shown by NBT and WST-1 assay. Thus, UVA-induced ROS, especially singlet oxygen and superoxide onion radicals were suggested to be scavenged by hydrogen and result in cytoprotection against ROS-induced mitochondrial dysfunction.

Similar results have been reported from previous research works on reconstruction of collagen in other dermal or epidermal cells by HW [38,39]. As a mechanism for using HW to treat PU at aspect of dermal and epidermal cells, we consider that there are three pathways as follows: (1) the promotion of the formation of dermis structure as well as reconstruction to type-I collagen, (2) the prevention of the formation of wound granules on disintegrated necrosis loci, and (3) the repair and restoration of scar tissues.

Healing effects for PU patients through HW intake via TF as shown by our present study have been scarcely found in the past. Our experience in this study added further evidences to a possible role in medical therapies for PU. Additionally, as well known, there are different methods to manufacture the hydrogen water by diverse research groups, so there are also different water-parameters about HW. In order to show our data obtained from measurements with the different dilution ratios, we had specially set up Figures 1 and 2, as well as Table 2 to present these achievements. How to manufacture HW and RW is an important and essential matter in the field of hydrogen water medicine.

But, this study has some limitations that should be considered when interpreting the results. Firstly the study design could not be carried out as the randomized control trail (RCT), because generally PU-cure clinical intervention test cannot be executed as RCT owing to other diverse factors such as various diseases concurrence and complication. Clinical situation did not allow us to get clinical data prior to one as we designed. Secondly we could not design the trial into comparing the results both HW oral intake and external washing of injurious sites with HW, respectively. These deserve the next-step study.

## Conclusions

HW intake via TF was demonstrated, for severely hospitalized elderly patients with PU, to execute wound size reduction and early recovery, both of which potently ensue from either type-I collagen construction in dermal fibroblasts or the promoted mitochondrial reducing ability and ROS repression in epidermal keratinocytes as shown by immunostain, NBT and WST-1 assays, respectively.

## Consent

Written informed consents that were presented from the patients for the publication of this report and any accompanying images were obtained and confirmed as the ethical clearance by the Ethics Committee of Kobayashi Hospital, Fukuyama City, Hiroshima Prefecture, Japan.

## Competing interests

The authors declare that they have no competing interests.

## Authors’ contributions

Conducted and designed the experiments: QL NM. Performed the experiments: DM SK QL. Analyzed the data: QL NM. Contributed apparatus/reagents/materials: DM HT NM. Wrote the manuscript: QL NM. All authors read and approved the final manuscript.
